# Supplementary material for: Evaluating the impact of COVID-19 pandemic-related home confinement on the refractive error of school-aged children in Germany: a cross-sectional study based on data from 414 eye care professional centres
Source: BMJ Open. 2023 Nov 21;13(11):e071833. doi: 10.1136/bmjopen-2023-071833 (PMC10668271; doi:10.1136/bmjopen-2023-071833)
Supplement: Supplementary data [file bmjopen-2023-071833supp001.pdf]

Supplemental table 1. Mean annual spherical equivalent refraction (SER) values based on individual age groups.

Age (years)	Sample size	2015	2016	2017	2018	2019	2020	2021	p value (2020) [a]	p value (2021) [b]
**6**	5579	1.11(0.06)	1.16(0.06)	1.27(0.07)	1.04(0.06)	1.18(0.05)	1.12(0.07)	0.97(0.08)	0.80	0.03
**7**	5540	0.73(0.06)	0.75(0.06)	0.74(0.06)	0.79(0.05)	0.81(0.06)	0.71(0.06)	0.68(0.07)	0.96	0.32
**8**	5700	0.41(0.06)	0.42(0.06)	0.37(0.06)	0.45(0.06)	0.51(0.06)	0.27(0.06)	0.24(0.07)	0.09	0.005
**9**	5779	-0.01(0.06)	0.03(0.06)	-0.02(0.06)	0.10(0.06)	0.06(0.06)	-0.12(0.06)	-0.14(0.06)	0.11	<0.001
**10**	6272	-0.27(0.05)	-0.22(0.05)	-0.19(0.06)	-0.28(0.05)	-0.23(0.05)	-0.43(0.06)	-0.41(0.07)	0.001	<0.001
**11**	6009	-0.43(0.06)	-0.38(0.06)	-0.42(0.06)	-0.30(0.06)	-0.25(0.06)	-0.43(0.06)	-0.49(0.06)	0.44	<0.001
**12**	6068	-0.64(0.06)	-0.52(0.06)	-0.69(0.06)	-0.65(0.06)	-0.57(0.06)	-0.58(0.06)	-0.67(0.06)	0.64	0.29
**13**	6192	-0.67(0.05)	-0.66(0.06)	-0.73(0.06)	-0.71(0.06)	-0.72(0.06)	-0.83(0.06)	-0.83(0.06)	0.01	0.01
**14**	6262	-0.98(0.05)	-0.79(0.06)	-0.97(0.06)	-0.76(0.06)	-0.86(0.06)	-0.86(0.06)	-0.97(0.07)	0.42	0.18
**15**	6525	-0.89(0.05)	-0.86(0.06)	-0.86(0.05)	-0.90(0.05)	-0.96(0.06)	-0.93(0.06)	-0.95(0.06)	0.83	0.19

For each year, the values shown are mean SER and standard error of the mean. Between parentheses, the standard error of the mean. Values are given in diopters.

[a] p value associated with the comparison between SER values in 2020 and the averaged SER values among 2015-2019 for each age.
[b] p value associated with the comparison between SER values in 2021 and the averaged SER values among 2015-2019 for each age.
