# Supplementary material for: Evaluating the impact of COVID-19 pandemic-related home confinement on the refractive error of school-aged children in Germany: a cross-sectional study based on data from 414 eye care professional centres
Source: BMJ Open. 2023 Nov 21;13(11):e071833. doi: 10.1136/bmjopen-2023-071833 (PMC10668271; doi:10.1136/bmjopen-2023-071833)
Supplement: Supplementary data [file bmjopen-2023-071833supp002.pdf]

Supplemental table 2. Percentage of myopes as a function of age (6-15) and year (2015-2021).

<table>
<tr><th>Age (years)</th><th>Sample size</th><th>2015</th><th>2016</th><th>2017</th><th>2018</th><th>2019</th><th>2020</th><th>2021</th><th>p value (2020) [a]</th><th>Z value (2020) [b]</th><th>p value (2021) [c]</th><th>Z value (2021) [d]</th><th>p value (2020) [e]</th><th>Z value (2020) [f]</th><th>p value (2021) [g]</th><th>Z value (2021) [h]</th><th>p value (2020) [i]</th><th>Z value (2020) [j]</th><th>p value (2021) [k]</th><th>Z value (2021) [l]</th></tr>
<tr><td>6</td><td>5579</td><td>10.92</td><td>12.27 [m]</td><td>12.18</td><td>9.97</td><td>10.51</td><td>12.16</td><td>16.09</td><td>0.94</td><td>0.07</td><td>0.03</td><td>-2.19</td><td>0.54</td><td>-0.61</td><td>0.005</td><td>-2.83</td><td rowspan="10">0.25</td><td rowspan="10">-1.16</td><td rowspan="10">0.002</td><td rowspan="10">-3.15</td></tr>
<tr><td>7</td><td>5540</td><td>20.67 [m]</td><td>18.10</td><td>18.82</td><td>17.68</td><td>18.35</td><td>20.47</td><td>21.07</td><td>0.92</td><td>0.10</td><td>0.84</td><td>-0.20</td><td>0.39</td><td>-0.87</td><td>0.25</td><td>-1.16</td></tr>
<tr><td>8</td><td>5700</td><td>29.44</td><td>27.88</td><td>30.90 [m]</td><td>26.65</td><td>27.17</td><td>32.00</td><td>35.82</td><td>0.62</td><td>-0.49</td><td>0.03</td><td>-2.12</td><td>0.11</td><td>-1.59</td><td>0.002</td><td>-3.17</td></tr>
<tr><td>9</td><td>5779</td><td>43.72 [m]</td><td>40.94</td><td>43.52</td><td>42.17</td><td>38.46</td><td>44.35</td><td>48.09</td><td>0.80</td><td>-0.26</td><td>0.07</td><td>-1.80</td><td>0.30</td><td>-1.06</td><td>0.01</td><td>-2.58</td></tr>
<tr><td>10</td><td>6272</td><td>50.85</td><td>50.67</td><td>51.50 [m]</td><td>50.67</td><td>49.43</td><td>54.67</td><td>57.25</td><td>0.19</td><td>-1.31</td><td>0.02</td><td>-2.40</td><td>0.09</td><td>-1.68</td><td>0.005</td><td>-2.78</td></tr>
<tr><td>11</td><td>6009</td><td>56.32</td><td>53.96</td><td>56.41 [m]</td><td>52.20</td><td>52.17</td><td>53.02</td><td>59.03</td><td>0.17</td><td>1.39</td><td>0.29</td><td>-1.07</td><td>0.62</td><td>0.49</td><td>0.04</td><td>-1.98</td></tr>
<tr><td>12</td><td>6068</td><td>61.15</td><td>58.55</td><td>63.07</td><td>63.32 [m]</td><td>59.18</td><td>61.49</td><td>60.48</td><td>0.44</td><td>0.77</td><td>0.24</td><td>1.17</td><td>0.86</td><td>-0.18</td><td>0.81</td><td>0.24</td></tr>
<tr><td>13</td><td>6192</td><td>65.40 [m]</td><td>61.91</td><td>63.41</td><td>63.53</td><td>62.93</td><td>67.76</td><td>67.43</td><td>0.29</td><td>-1.06</td><td>0.37</td><td>-0.89</td><td>0.06</td><td>-1.91</td><td>0.09</td><td>-1.72</td></tr>
<tr><td>14</td><td>6262</td><td>70.81 [m]</td><td>66.74</td><td>68.88</td><td>66.25</td><td>67.91</td><td>69.07</td><td>69.45</td><td>0.43</td><td>0.79</td><td>0.54</td><td>0..62</td><td>0.67</td><td>-0.42</td><td>0.55</td><td>-0.60</td></tr>
<tr><td>15</td><td>6525</td><td>67.98</td><td>67.62</td><td>69.92</td><td>68.42</td><td>70.31 [m]</td><td>66.86</td><td>70.22</td><td>0.12</td><td>1.56</td><td>0.97</td><td>0.04</td><td>0.36</td><td>0.91</td><td>0.53</td><td>-0.63</td></tr>
</table>

Myopia is defined as SER ≤ −0.50D. Values are expressed in percent. P values are calculated based on two proportion Z-test.

[a] p value associated with the comparison between the percentage of myopes in 2020 and the highest percentage in 2015-2019.

[b] Z statistic value associated with the comparison between the percentage of myopes in 2020 and the highest percentage in 2015-2019.

[c] p value associated with the comparison between the percentage of myopes in 2021 and the highest percentage in 2015-2019.

[d] Z statistic value associated with the comparison between the percentage of myopes in 2021 and the highest percentage in 2015-2019.

[e] p value associated with the comparison between the percentage of myopes in 2020 and the averaged percentage among 2015-2019.

[f] Z statistic value associated with the comparison between the percentage of myopes in 2020 and the averaged percentage among 2015-2019.

[g] p value associated with the comparison between the percentage of myopes in 2021 and the averaged percentage among 2015-2019.

[h] Z statistic value associated with the comparison between the percentage of myopes in 2021 and the averaged percentage among 2015-2019.

[i] p value associated with the comparison between the percentage of myopes in 2020 and the averaged percentage among 2015-2019 for all ages.

[j] Z statistic value associated with the comparison between the percentage of myopes in 2020 and the averaged percentage among 2015-2019 for all ages.

[k] p value associated with the comparison between the percentage of myopes in 2021 and the averaged percentage among 2015-2019 for all ages.

[l] Z statistic value associated with the comparison between the percentage of myopes in 2021 and the averaged percentage among 2015-2019 for all ages.

[m] Highest percentage of myopia relative to each age group, within the year range 2015-2019.
