# Supplementary material for: Evaluating the impact of COVID-19 pandemic-related home confinement on the refractive error of school-aged children in Germany: a cross-sectional study based on data from 414 eye care professional centres
Source: BMJ Open. 2023 Nov 21;13(11):e071833. doi: 10.1136/bmjopen-2023-071833 (PMC10668271; doi:10.1136/bmjopen-2023-071833)
Supplement: Supplementary data [file bmjopen-2023-071833supp003.pdf]

Supplemental table 3. Sample sizes as a function of age, gender, and year.

Age (years)	Gender	2015	2016	2017	2018	2019	2020	2021
6	female	394	407	433	427	464	355	363
	male	375	457	396	365	402	377	364
7	female	482	424	417	441	373	399	375
	male	384	399	396	385	390	324	351
8	female	453	439	460	403	423	440	407
	male	369	393	417	370	372	385	369
9	female	476	453	466	425	448	446	453
	male	400	380	375	360	371	368	358
10	female	573	534	513	521	512	464	464
	male	424	439	388	377	360	339	364
11	female	537	503	510	473	533	474	450
	male	413	407	348	345	343	337	336
12	female	534	567	564	509	533	523	472
	male	367	374	354	317	344	295	315
13	female	589	520	619	553	512	542	483
	male	356	349	373	341	335	314	306
14	female	568	552	554	607	541	475	465
	male	405	356	394	347	347	314	337
15	female	563	642	633	591	567	500	515
	male	399	374	381	362	349	348	301
