# Supplementary material for: Evaluating the impact of COVID-19 pandemic-related home confinement on the refractive error of school-aged children in Germany: a cross-sectional study based on data from 414 eye care professional centres
Source: BMJ Open. 2023 Nov 21;13(11):e071833. doi: 10.1136/bmjopen-2023-071833 (PMC10668271; doi:10.1136/bmjopen-2023-071833)
Supplement: Author's manuscript [file bmjopen-2023-071833.draft_revisions.pdf]

BMJ Open is committed to open peer review. As part of this commitment we make the peer review history of every article we publish publicly available.

When an article is published we post the peer reviewers' comments and the authors' responses online. We also post the versions of the paper that were used during peer review. These are the versions that the peer review comments apply to.

The versions of the paper that follow are the versions that were submitted during the peer review process. They are not the versions of record or the final published versions. They should not be cited or distributed as the published version of this manuscript.

BMJ Open is an open access journal and the full, final, typeset and author-corrected version of record of the manuscript is available on our site with no access controls, subscription charges or pay-per-view fees (http://bmjopen.bmj.com).

If you have any questions on BMJ Open's open peer review process please email info.bmjopen@bmj.com

BMJ Open

Impact of COVID-19 pandemic-related home confinement on the refractive error of school-aged children in Germany

Journal:	*BMJ Open*
Manuscript ID	bmjopen-2023-071833
Article Type:	Original research
Date Submitted by the Author:	12-Jan-2023
Complete List of Authors:	Sanz Diez, Pablo; Carl Zeiss Vision GmbH, Technology and Innovation; Eberhard Karls Universitat Tubingen, Institute for Ophthalmic Research<br>Ohlendorf, Arne; Carl Zeiss Vision GmbH, Technology and Innovation; Eberhard Karls Universitat Tubingen, Institute for Ophthalmic Research<br>Barraza-Bernal, Maria Jose; Carl Zeiss Vision GmbH, Technology and Innovation<br>Kratzer, Timo; Carl Zeiss Vision GmbH, Technology and Innovation<br>Wahl, Siegfried; Carl Zeiss Vision GmbH, Technology and Innovation; Eberhard Karls Universitat Tubingen, Institute for Ophthalmic Research
Keywords:	COVID-19, Public health < INFECTIOUS DISEASES, Paediatric ophthalmology < OPHTHALMOLOGY

SCHOLARONE™
Manuscripts

I, the Submitting Author has the right to grant and does grant on behalf of all authors of the Work (as defined in the below author licence), an exclusive licence and/or a non-exclusive licence for contributions from authors who are: i) UK Crown employees; ii) where BMJ has agreed a CC-BY licence shall apply, and/or iii) in accordance with the terms applicable for US Federal Government officers or employees acting as part of their official duties; on a worldwide, perpetual, irrevocable, royalty-free basis to BMJ Publishing Group Ltd ("BMJ") its licensees and where the relevant Journal is co-owned by BMJ to the co-owners of the Journal, to publish the Work in this journal and any other BMJ products and to exploit all rights, as set out in our licence.

The Submitting Author accepts and understands that any supply made under these terms is made by BMJ to the Submitting Author unless you are acting as an employee on behalf of your employer or a postgraduate student of an affiliated institution which is paying any applicable article publishing charge ("APC") for Open Access articles. Where the Submitting Author wishes to make the Work available on an Open Access basis (and intends to pay the relevant APC), the terms of reuse of such Open Access shall be governed by a Creative Commons licence – details of these licences and which Creative Commons licence will apply to this Work are set out in our licence referred to above.

Other than as permitted in any relevant BMJ Author's Self Archiving Policies, I confirm this Work has not been accepted for publication elsewhere, is not being considered for publication elsewhere and does not duplicate material already published. I confirm all authors consent to publication of this Work and authorise the granting of this licence.

For peer review only

Impact of COVID-19 pandemic-related home confinement on the refractive error of school-aged children in Germany

Pablo Sanz Diez, PhD [1, 3 *]; Arne Ohlendorf, PhD [1, 3]; Maria Barraza-Bernal, PhD [2]; Timo Kratzer [2]; Siegfried Wahl, Prof. [1,3]

1. Carl Zeiss Vision International GmbH, Technology & Innovation, Aalen, Germany.
2. Carl Zeiss Vision GmbH, Technology & Innovation, Aalen, Germany.
3. Institute for Ophthalmic Research, Eberhard Karls University Tuebingen, Tuebingen, Germany.

Corresponding author's contact information:

Pablo Sanz Diez

Carl Zeiss Vision International GmbH, Technology & Innovation

Institute for Ophthalmic Research, Eberhard Karls University Tuebingen

Elfriede-Aulhorn-Strasse 7

72076 Tuebingen, Germany

Phone: + 49 7071 29 80810

pablo.sanzdiez@zeiss.com

For peer review only

Abstract

Objective: This study aimed at evaluating refractive changes in German school-aged children before and after the COVID-19 pandemic.

Design: Cross-sectional study.

Setting: 414 eye care professional centers from Germany.

Participants: Refractive data from 59926 German children aged 6 to 15 years were examined over a 7-year period (2015-2021).

Primary and secondary outcome measures: Spherical equivalent refraction was assessed as a function of year, age, and gender. The refractive values concerning 2020 and 2021 were compared with those assigned to prior years (2015-2019).

Results: The refractive data associated with 2020 and 2021 showed a myopic refractive shift of approximately -0.20D compared to the 2015-2019 range. The refractive change was statistically considerable in the 6- to 11-year range ($p<0.05$), while from 12 to 15 years was negligible ($p\geq0.10$). Percentage of myopes was also impacted in 2021 ($p=0.002$), but not in 2020 ($p=0.25$). From 6 to 11 years, the percentage of myopes in 2021 increased significantly by 6.02% compared to the 2015–2019 range ($p\leq0.04$). The highest percentage increase occurred at 8 and 10 years of age, showing a rise of 7.42% ($p=0.002$) and 6.62% ($p=0.005$), respectively. From 12 to 15 years, there was no significant increase in the percentage of myopes in 2021 ($p\geq0.09$). Percentage of myopes in 2020 was not influenced at any age ($p\geq0.06$).

Conclusion: Disruption of normal lifestyle due to pandemic-related home confinement appears to lead to a myopic refractive shift in children aged 6 to 11 years in Germany. The greater effect observed at younger ages seems to emphasize the importance of refractive development in this age group.

For peer review only

Strengths and limitations of this study

- This is the first study aimed at reporting the impact of COVID-19 pandemic-related home confinement on the refractive error of school-aged children in Germany.
- Data were collected from a network management software from a total of 414 eye care professional centers in Germany. Data from such a diverse set of centers increases the representativeness of the sample and the generalizability of study's findings making them more applicable to a wider population.
- Refraction data analysis of 7-year period, from 2015-2021. The current study is one of the few studies that includes data from 2021 to explore the effect of COVID-19 pandemic-related home confinement on the refractive error of school-aged children. Such long-term data provide valuable insights into how home confinement during the pandemic may have affected the refractive error of these children over time, as well as identifying any potential trends or patterns.
- The nature of the database collected by a network management software for eye care professional centers may limit additional information that could help to better understand and interpret the obtained results.

For peer review only

Introduction

Coronavirus disease 2019 (COVID-19), characterized as a pandemic by the World Health Organization on March 11, 2020,[1] has resulted in a global health, social, and economic crisis.[2 3] Containment and control of the disease was the priority strategy adopted by most governments. Mandatory use of masks, social distancing, self-isolation, and nationwide home confinement were the main measures aimed at curbing the spread of the disease.[4] In many countries, the home confinement policy has restricted citizens from leaving their homes except for justified reasons, which has led to many negative psychosocial and psychological consequences.[5-7] Regarding ocular health, the increased time spent indoors and working on near work activities, such as screen time, reading, writing, among many others, both linked to myopia development,[8 9] has raised interest about the impact of home confinement on the refractive status of school-aged children. In fact, changes in the mean spherical equivalent refraction and increased myopia prevalence have already been clearly reported as collateral consequences in school-aged children.[10-17] The purpose of the current study was to assess whether COVID-19 pandemic-related home confinement caused refractive changes in school-aged children in Germany.

Methods

Study dataset

The dataset used for the analyses described in the current study were obtained from Euronet (Euronet Market Research, Euronet Software AG, Frechen, Germany). Euronet is a network management software for eye care centers, which has been administering clinical and ocular history data in Germany since 2001.

For peer review only

For the current study, the dataset was comprised of the following variables: center identification number, subject identification number, purchase date, date of birth, age, gender, spectacle-plane refractive correction (sphere, cylinder, axis), and visual acuity.

Study population

First-visit refractive data from 67137 children aged 6 to 15 years were collected from 414 eye care professional (ECP) centers in Germany between 2015 and 2021.

For the final analysis, 34300 females and 25626 males were considered. A total of 7211 cases were excluded due to incomplete gender information.

The present study was in accordance with the declaration of Helsinki. All data involved in the current research were de-identified and protected by the privacy safeguards of the European General Data Protection Regulation.

Statistical Analysis

Statistical analyses were conducted under the MATLAB R2020a statistics and machine learning toolbox (MathWorks, Massachusetts, USA). Statistical tests were chosen according to the study purposes and data distribution, which was previously assessed by means of the Kolmogorov-Smirnov test. Differences in the yearly refractive error were examined by two-sided Mann–Whitney U test or Kruskal-Wallis test followed by Tukey-Kramer post-hoc test. A two-proportion Z-test was used to determine statistical differences in the percentage of myopes among the evaluated years. Differences were considered statistically significant when $p<0.05$.

All values shown here represent spherical equivalent refraction (SER) data. SER values were calculated as the sum of the sphere power with half of the negative cylinder power. The purchase date was the variable considered to establish the SER values as a function of year. Only the data from the right eye was used for the final analysis. For the percentage of myopes assessment, myopia was defined as SER ≤ -0.50D.

For peer review only

Patient and public involvement

None of the individuals were involved in the design, implementation, reporting or dissemination plans of our research.

Results

Data from a total of 59926 German children aged 6 to 15 years were evaluated in this study. 34300 females (57.24%; mean age: 10.82±2.84 years; and mean SER: -0.22±1.79D) and 25626 males (42.76%; mean age: 10.40±2.88 years; and mean SER: -0.15±1.93D) comprised the reported outcomes. Among the two gender groups, the mean SER values was -0.19±1.85D, while the minimum and maximum SER values were -24.25 and 17.50D, respectively. The most positive mean SER was found in the 6-year age group (1.12±1.78D), while the most negative mean SER among children by age of 15 (-0.91±1.68D). On average, SER was age-dependent (χ^2=6250.63, p<0.001; and χ^2=4676.7, p<0.001, for females and males respectively), and gender-dependent for specific age groups. From 6 to 11 years, both gender groups showed similar SER values (p≥0.07), whereas from 12 to 15 years, males exhibited more negative SER values compared to females in their respective age groups (p<0.02).

Figure 1 and Table 1 show the SER distribution and mean SER values for individual age groups along a 7-year period (2015-2021). On average, among all years, 2020 and 2021 presented the highest negative SER values, being 2021 the most statistically evident (Table 1). From 2015 to 2019, mean SER values remain stable towards more positive readings. A comparison of 2020 and 2021 with previous years revealed a 0.2D myopic shift in those children aged 6 to 11 years (p<0.05). Between 12 to 15 years, the myopic shift in 2020 and 2021 was less than 0.08D (p≥0.10).

For peer review only

Table 1. Mean annual spherical equivalent refraction (SER) values based on individual age groups.

Age (years)	Sample size	2015	2016	2017	2018	2019	2020	2021	p value (2020) [a]	p value (2021) [b]
**6**	5579	1.11(0.06)	1.16(0.06)	1.27(0.07)	1.04(0.06)	1.18(0.05)	1.12(0.07)	0.97(0.08)	0.80	0.03
**7**	5540	0.73(0.06)	0.75(0.06)	0.74(0.06)	0.79(0.05)	0.81(0.06)	0.71(0.06)	0.68(0.07)	0.96	0.32
**8**	5700	0.41(0.06)	0.42(0.06)	0.37(0.06)	0.45(0.06)	0.51(0.06)	0.27(0.06)	0.24(0.07)	0.09	0.005
**9**	5779	-0.01(0.06)	0.03(0.06)	-0.02(0.06)	0.10(0.06)	0.06(0.06)	-0.12(0.06)	-0.14(0.06)	0.11	<0.001
**10**	6272	-0.27(0.05)	-0.22(0.05)	-0.19(0.06)	-0.28(0.05)	-0.23(0.05)	-0.43(0.06)	-0.41(0.07)	0.001	<0.001
**11**	6009	-0.43(0.06)	-0.38(0.06)	-0.42(0.06)	-0.30(0.06)	-0.25(0.06)	-0.43(0.06)	-0.49(0.06)	0.44	<0.001
**12**	6068	-0.64(0.06)	-0.52(0.06)	-0.69(0.06)	-0.65(0.06)	-0.57(0.06)	-0.58(0.06)	-0.67(0.06)	0.64	0.29
**13**	6192	-0.67(0.05)	-0.66(0.06)	-0.73(0.06)	-0.71(0.06)	-0.72(0.06)	-0.83(0.06)	-0.83(0.06)	0.01	0.01
**14**	6262	-0.98(0.05)	-0.79(0.06)	-0.97(0.06)	-0.76(0.06)	-0.86(0.06)	-0.86(0.06)	-0.97(0.07)	0.42	0.18
**15**	6525	-0.89(0.05)	-0.86(0.06)	-0.86(0.05)	-0.90(0.05)	-0.96(0.06)	-0.93(0.06)	-0.95(0.06)	0.83	0.19

For each year, the values shown are mean SER and standard error of the mean. Between parentheses, the standard error of the mean. Values are given in diopters.

[a] p value associated with the comparison between SER values in 2020 and the averaged SER values among 2015-2019 for each age.

[b] p value associated with the comparison between SER values in 2021 and the averaged SER values among 2015-2019 for each age.

Analyzing the data by three age ranges evidenced 2021 as the year with the highest myopic mean SER values (Figure 2). As seen in Figure 2, considering 2021 as benchmark revealed maximum SER differences of -0.22D (95% CI: -0.37 to -0.07; p<0.001), -0.20D (95% CI: -0.34 to -0.05; p<0.001) and -0.17D (95% CI: -0.31 to -0.02; p<0.001) in the age ranges 6 to 9, 9 to 12, and 12 to 15, respectively. 9 to 12 age range showed statistically significant differences among 2021 and all previous years, from 2015 to 2019 (χ^2=49.41, p<0.001; post-hoc: p<0.004 all). Smaller SER differences were found by comparing 2020 and prior years (Figure 2). Here, 2020 exhibited maximum SER differences of -0.15D (95% CI: -0.31 to 0.00; p=0.04), -0.18D (95% CI: -0.33 to -0.04; p<0.01) and -0.10D (95% CI: -0.25 to 0.04; p<0.01) for 6 to 9, 9 to 12, and 12 to 15 age ranges, respectively.

For peer review only - http://bmjopen.bmj.com/site/about/guidelines.xhtml

The myopic shift observed in 2021 seems to be followed by an increase in the percentage of myopes in children aged 6 to 11 years (Table 2). On average, in this age range, the percentage of myopes in 2021 increased significantly by 6.02% compared to the 2015–2019-year range ($p \leq 0.04$; $Z \leq .49$). The highest increase in the percentage of myopes was found at 8 and 10 years of age, showing a rise of 7.42% ($p=0.002$; $Z=-3.17$) and 6.62% ($p=0.005$; $Z=-2.78$), respectively. From 12 to 15 years, no significant changes in the percentage of myopes in 2021 were found ($p \geq 0.09$; $Z \geq -1.72$). Instead, as seen in Table 2, no significant changes in the percentage of myopes at any age were noted in 2020 relative to the 2015-2019 range ($p=0.25$; $Z=-1.16$ and $p \geq 0.06$; $Z \geq -1.91$ for all ages).

For peer review only

Table 2. Percentage of myopes as a function of age (6-15) and year (2015-2021).

Age (years)	Sample size	2015	2016	2017	2018	2019	2020	2021	p value (2020) [a]	Z value (2020) [b]	p value (2021) [c]	Z value (2021) [d]	p value (2020) [e]	Z value (2020) [f]	p value (2021) [g]	Z value (2021) [h]	p value (2020) [i]	Z value (2020) [j]	p value (2021) [k]	Z value (2021) [l]
**6**	5579	10.92	12.27 [m]	12.18	9.97	10.51	12.16	16.09	0.94	0.07	0.03	-2.19	0.54	-0.61	0.005	-2.83	0.25	-1.16	0.002	-3.15
**7**	5540	20.67 [m]	18.10	18.82	17.68	18.35	20.47	21.07	0.92	0.10	0.84	-0.20	0.39	-0.87	0.25	-1.16				
**8**	5700	29.44	27.88	30.90 [m]	26.65	27.17	32.00	35.82	0.62	-0.49	0.03	-2.12	0.11	-1.59	0.002	-3.17				
**9**	5779	43.72 [m]	40.94	43.52	42.17	38.46	44.35	48.09	0.80	-0.26	0.07	-1.80	0.30	-1.06	0.01	-2.58				
**10**	6272	50.85	50.67	51.50 [m]	50.67	49.43	54.67	57.25	0.19	-1.31	0.02	-2.40	0.09	-1.68	0.005	-2.78				
**11**	6009	56.32	53.96	56.41 [m]	52.20	52.17	53.02	59.03	0.17	1.39	0.29	-1.07	0.62	0.49	0.04	-1.98				
**12**	6068	61.15	58.55	63.07	63.32 [m]	59.18	61.49	60.48	0.44	0.77	0.24	1.17	0.86	-0.18	0.81	0.24				
**13**	6192	65.40 [m]	61.91	63.41	63.53	62.93	67.76	67.43	0.29	-1.06	0.37	-0.89	0.06	-1.91	0.09	-1.72				
**14**	6262	70.81 [m]	66.74	68.88	66.25	67.91	69.07	69.45	0.43	0.79	0.54	0..62	0.67	-0.42	0.55	-0.60				
**15**	6525	67.98	67.62	69.92	68.42	70.31 [m]	66.86	70.22	0.12	1.56	0.97	0.04	0.36	0.91	0.53	-0.63				

Myopia is defined as SER ≤ −0.50D. Values are expressed in percent. P values are calculated based on two proportion Z-test.

[a] p value associated with the comparison between the percentage of myopes in 2020 and the highest percentage in 2015-2019.

[b] Z statistic value associated with the comparison between the percentage of myopes in 2020 and the highest percentage in 2015-2019.

[c] p value associated with the comparison between the percentage of myopes in 2021 and the highest percentage in 2015-2019.

[d] Z statistic value associated with the comparison between the percentage of myopes in 2021 and the highest percentage in 2015-2019.

[e] p value associated with the comparison between the percentage of myopes in 2020 and the averaged percentage among 2015-2019.

[f] Z statistic value associated with the comparison between the percentage of myopes in 2020 and the averaged percentage among 2015-2019.

[g] p value associated with the comparison between the percentage of myopes in 2021 and the averaged percentage among 2015-2019.

[h] Z statistic value associated with the comparison between the percentage of myopes in 2021 and the averaged percentage among 2015-2019.

[i] p value associated with the comparison between the percentage of myopes in 2020 and the averaged percentage among 2015-2019 for all ages.

[j] Z statistic value associated with the comparison between the percentage of myopes in 2020 and the averaged percentage among 2015-2019 for all ages.

[k] p value associated with the comparison between the percentage of myopes in 2021 and the averaged percentage among 2015-2019 for all ages.

[l] Z statistic value associated with the comparison between the percentage of myopes in 2021 and the averaged percentage among 2015-2019 for all ages.

[m] Highest percentage of myopia relative to each age group, within the year range 2015-2019.

For peer review only

Gender-specific effects were depicted in Figure 3. When comparing 2021 with the 2015-2019 range, both genders showed similar refractive trends. The largest refractive differences occurred between 6 and 11 years of age (Figure 3). Here, 2021 showed a mean refractive shift of -0.17D and -0.16D for females and males, respectively. Within the range 2015-2019, 2019 was the year with the biggest refractive differences relative to 2021. Females experienced the greatest refractive shift from 6 to 11 years (-0.27D), while for males it was among 8 and 13 years (-0.21D).

For peer review only

Discussion

In the present study we have shown refractive data concerning a period of 7 consecutive years (2015-2021) in a population of children aged 6 to 15 years in Germany. In the 2015-2019 range, the mean SER values showed stable tendencies with no meaningful variations among them. However, in 2020 and 2021, we found a shift in SER toward more myopic values when compared to previous years (Figure 1, Figure 2, and Table 1). Interestingly, the refractive shift appeared to be dependent on the population age. From 6 to 11 years, children showed a significant mean refractive shift of approximately -0.20D, while from 12 to 15 years, the mean change was smaller than -0.08D.

Examining the 2020 and 2021 refractive data, 2021 was revealed as the year with the most prominent shift. All these refractive trends were observed in both males and females, with the 6- to 11-year-old range again being the most influenced.

Percentage of myopes was also affected, however, only in 2021. Again, the statistically significant changes were noted in the 6- to 11-year range. Here, the percentage of myopes in 2021 was on average 6% higher than in the 2015-2019 range. In contrast, no significant changes in the percentage of myopes were observed in 2020. These findings reinforce 2021 as the most impacted year.

Our findings agree with those studies reporting the impact of COVID-19 home confinement on myopia development in schoolchildren.[10-17] Wang J et al,[10] from 2015 to 2020, measured the refractive data of 123535 children (59200 females and 64335 males) aged 6 to 13 years from ten primary schools in Feicheng, China. Authors found that children aged 6 to 8 years exhibited a mean myopic shift of -0.30D, while those aged 9 to 13 years less than -0.10D. A 1.4 to 3-fold increase in myopia prevalence was also noted in children aged 6 to 8 years (15.8% for 6 years, 10% for 7 years, and 9.5% for 8 years). No significant increase was detected in 9- to 13-year-olds.[10] Other Asian population-based studies revealed similar results. Xu L et al,[12] observed a

For peer review only

mean increase in myopia prevalence of 6.50%, which was school-grade dependent, 8.54% (from grades 1 to 6) and 4.32% (from grades 7 to 12). Myopia progression increased 1.5 times and was faster in the youngest, grades 1 to 6.[12] Hu Y et al,[11] found that grade 3 students experienced a myopic SER shift of -0.35D and a myopia prevalence increase of 7.5%. Ma D et al,[17] found a -0.6D change in SER in children aged 8 to 10 years following 7-month period of home study during the pandemic. A similar refractive change was reported by Ma M et al[16] after a refractive screening of 201 myopic children aged 7 to 12 years during the period from April 2019 to May 2020. In May 2020, a refractive shift of -0.59D was determined.[16] Yang X et al[15] found a myopic SER change but only in low hyperopia, emmetropia, and mild myopia in school grades 1-4 and 7. On average, between 2019 and 2020, the reported SER change was -0.39D.[15]

In Europe, few studies have evaluated the effect of pandemic home confinement on children's refractive development. A cross-sectional study in Spanish children aged 5 to 7 years reported a mean SER shift of -0.18 in 2020 compared with 2019. In their study population, the myopia percentage remained stable, while hyperopia and emmetropia decreased and increased, respectively.[14] Likewise, in 2021, Italian children aged 5 to 12 years showed a mean SER reduction of -0.50D and a mean increase in myopia prevalence of 9.12%. Those aged 9 to 12 years were the most influenced.[13]

These findings, in both Asian and European populations, are consistent with ours. Overall, after the pandemic home confinement period, we have observed a SER myopic shift and an increase in the percentage of myopes in the studied German school-aged population. As described in prior studies,[10 12 13 15] the refractive aftereffects established in our study were also age dependent. While no effect was seen from 12 to 15 years of age, the most important refractive changes occurred in the age range of 6 to 11 years. Specifically, the highest refractive and myopia percentage changes appeared at ages 8 to 11 years, compatible with the age range described in Asian[10 12 15] and Italian[13] children. As postulated by Wang et al,[10] younger children seem to be more sensitive to environmental changes than older ones. This hypothesis is

For peer review only

supported by those studies that have already documented a faster myopia progression[18] and axial elongation[19] at younger ages. Apparently, the ocular and refractive plasticity involved at this age window may be crucial,[10] however further assessments are required to draw more robust assumptions.

As previously seen, the refractive and percentage change rates calculated in the current study were comparable but not strictly equal to those reported by other authors. Small refractive variations among studies may be due to multiple reasons: application of different refraction techniques, genetic predisposition of the study population, interaction of environmental factors or even different home confinement regulations. Perhaps the more restrictive nationwide confinement regulations established in China or Italy, compared to Germany, have made the refractive aftereffects even higher and already visible in 2020, as seen in the Asian population-based studies. As previously reported, in our study the refractive aftereffects were found to be more evident in 2021 rather than in 2020. The effects of certain myopia risk factors related to the pandemic, such as increased screen time, reduced outdoor time, disruptions to healthcare access, as well as increased stress and anxiety may take time to appear or to be detected in terms of myopia. For example, increased screen time and reduced outdoor time, due to COVID-19 restrictions, may not immediately lead to myopia, but may contribute to its development over time. Changes in healthcare access may also take time to impact myopia prevalence, as delays in diagnosis and treatment may not be immediately apparent. Stress and anxiety may not cause myopia directly but may exacerbate existing myopia or make it more difficult to manage. More research is needed to fully understand the time course of these effects and to identify any other potential contributing factors. It is also important to consider the impact of individual differences and environmental factors on the relationship between the COVID-19 pandemic and myopia. Additionally, it should be noted that most of the published studies on the refractive effects of COVID-19 pandemic-related home confinement did not analyze refractive data from 2021. It would be interesting to see if they also find a greater refractive effect in 2021 compared to 2020.

For peer review only

Undeniably, lifestyle behavioral changes induced by the COVID-19 pandemic have led to refractive changes in school-aged children. Government regulations such as home confinement have mainly resulted in increased near work activities, and reduced time spent outdoors, both risk factors for myopia.[8 9] All these results should warn governmental authorities when planning any nationwide lockdown with home confinement.[20] Further refractive studies over the next few years are needed to reveal whether this is a reversible outcome or not.

For peer review only

Limitations

The database used in the present study comes from a network management software for eye care centers. This database is anonymous and lacks certain information such as the type of refraction techniques used or the individual's ocular history data. Therefore, this raises certain study limitations. On one side, we cannot assure what kind of refraction techniques were used to obtain the refractive data. This aspect may introduce measurement errors that could influence our results. Nevertheless, we have used statistical methods that are robust to possible measurement errors. Additionally, the lack of information regarding ocular history data could also represent a limitation in our study. It is possible that some individuals with certain eye conditions, which could be considered as exclusion criteria, may have been included in the database. On the other side, our database lacked ocular biometric data such as axial length, anterior chamber depth, corneal curvature, and lens thickness. Lifestyle data, such as time spent outdoors and time in near work activities, were also missing.

Although the type of database used in the current study entails specific limitations, it also has numerous advantages. This type of database gave us access to a large sample size, which increases the statistical power of the study and provides a more robust analysis. Additionally, a database from such a diverse set of eye care professional centers increases the

representativeness of the sample and the generalizability of study's findings making them more applicable to a wider population.

Overall, despite the limitations, our results are consistent with prior research, and we believe that the current study provides useful data on the impact of COVID-19 pandemic-related home confinement on the refractive error of school-aged children in Germany.

Conclusion

Our study provides valuable insights into the impact of COVID-19 pandemic-related home confinement on the refractive error of school-aged children in Germany. In summary, our outcomes may associate home confinement with a myopic refractive shift in German children aged 6 to 11 years, according to the 2020 and 2021 refractive data. Interestingly, children aged 8 to 11 were the most affected, which seems to be consistent with the importance of that age window during myopia development.

For peer review only

Contributors: PSD, AO, MBB, TK, and SW conceived and planned the study. PSD, AO, and MBB performed the data analysis and data interpretation. TK and SW served as project manager for the study. PSD wrote the first draft of the manuscript. PSD AO, MBB, TK, and SW conducted the subsequent revisions. PSD, AO, MBB, TK, and SW provided technical and intellectual aspects for the study.

Competing interests: All authors declare no competing interests.

Funding: This research received no specific grant from any funding agency in the public, commercial or not-for-profit sectors.

Data sharing statement: Data are available upon reasonable request.

Ethics approval statement: The present study was in accordance with the declaration of Helsinki. All data involved in the current research were de-identified and protected by the privacy safeguards of the European General Data Protection Regulation.

For peer review only

References

1. World Health Organization. WHO Director-General's opening remarks at the media briefing on COVID-19-11 March 2020. Geneva, Switzerland 2020.
2. Pollard CA, Morran MP, Nestor-Kalinoski AL. The COVID-19 pandemic: a global health crisis. *Physiol Genomics* 2020;52(11):549-57. doi: 10.1152/physiolgenomics.00089.2020
3. Nicola M, Alsafi Z, Sohrabi C, et al. The socio-economic implications of the coronavirus pandemic (COVID-19): A review. *Int J Surg* 2020;78:185-93. doi: 10.1016/j.ijsu.2020.04.018
4. Farsalinos K, Poulas K, Kouretas D, et al. Improved strategies to counter the COVID-19 pandemic: Lockdowns vs. primary and community healthcare. *Toxicol Rep* 2021;8:1-9. doi: 10.1016/j.toxrep.2020.12.001
5. Rohr S, Muller F, Jung F, et al. [Psychosocial Impact of Quarantine Measures During Serious Coronavirus Outbreaks: A Rapid Review]. *Psychiatr Prax* 2020;47(4):179-89. doi: 10.1055/a-1159-5562
6. Brooks SK, Webster RK, Smith LE, et al. The psychological impact of quarantine and how to reduce it: rapid review of the evidence. *Lancet* 2020;395(10227):912-20. doi: 10.1016/S0140-6736(20)30460-8
7. Ahrens KF, Neumann RJ, Kollmann B, et al. Impact of COVID-19 lockdown on mental health in Germany: longitudinal observation of different mental health trajectories and protective factors. *Transl Psychiatry* 2021;11(1):392. doi: 10.1038/s41398-021-01508-2
8. Sherwin JC, Reacher MH, Keogh RH, et al. The association between time spent outdoors and myopia in children and adolescents: a systematic review and meta-analysis. *Ophthalmology* 2012;119(10):2141-51. doi: 10.1016/j.ophtha.2012.04.020
9. He M, Xiang F, Zeng Y, et al. Effect of Time Spent Outdoors at School on the Development of Myopia Among Children in China: A Randomized Clinical Trial. *JAMA* 2015;314(11):1142-8. doi: 10.1001/jama.2015.10803
10. Wang J, Li Y, Musch DC, et al. Progression of Myopia in School-Aged Children After COVID-19 Home Confinement. *JAMA Ophthalmol* 2021;139(3):293-300. doi: 10.1001/jamaophthalmol.2020.6239
11. Hu Y, Zhao F, Ding X, et al. Rates of Myopia Development in Young Chinese Schoolchildren During the Outbreak of COVID-19. *JAMA Ophthalmol* 2021;139(10):1115-21. doi: 10.1001/jamaophthalmol.2021.3563
12. Xu L, Ma Y, Yuan J, et al. COVID-19 Quarantine Reveals That Behavioral Changes Have an Effect on Myopia Progression. *Ophthalmology* 2021;128(11):1652-54. doi: 10.1016/j.ophtha.2021.04.001
13. Trovato Battagliola E, Mangiantini P, D'Andrea M, et al. Effect of COVID-19 lockdown on refractive errors in Italian children aged 5-12 years: A multi-center retrospective study. *Eur J Ophthalmol* 2022:11206721221106135. doi: 10.1177/11206721221106135
14. Alvarez-Peregrina C, Martinez-Perez C, Villa-Collar C, et al. Impact of COVID-19 Home Confinement in Children's Refractive Errors. *Int J Environ Res Public Health* 2021;18(10) doi: 10.3390/ijerph18105347
15. Yang X, Fan Q, Zhang Y, et al. Changes in Refractive Error Under COVID-19: A 3-Year Follow-up Study. *Adv Ther* 2022;39(6):2999-3010. doi: 10.1007/s12325-022-02150-0
16. Ma M, Xiong S, Zhao S, et al. COVID-19 Home Quarantine Accelerated the Progression of Myopia in Children Aged 7 to 12 Years in China. *Invest Ophthalmol Vis Sci* 2021;62(10):37. doi: 10.1167/iovs.62.10.37
17. Ma D, Wei S, Li SM, et al. Progression of myopia in a natural cohort of Chinese children during COVID-19 pandemic. *Graefes Arch Clin Exp Ophthalmol* 2021;259(9):2813-20. doi: 10.1007/s00417-021-05305-x

For peer review only

18. Jones-Jordan LA, Sinnott LT, Chu RH, et al. Myopia Progression as a Function of Sex, Age, and Ethnicity. *Invest Ophthalmol Vis Sci* 2021;62(10):36. doi: 10.1167/iovs.62.10.36
19. Sanz Diez P, Yang LH, Lu MX, et al. Growth curves of myopia-related parameters to clinically monitor the refractive development in Chinese schoolchildren. *Graefes Arch Clin Exp Ophthalmol* 2019;257(5):1045-53. doi: 10.1007/s00417-019-04290-6
20. Klaver CCW, Polling JR, Enthoven CA. 2020 as the Year of Quarantine Myopia. *JAMA Ophthalmol* 2021;139(3):300-01. doi: 10.1001/jamaophthalmol.2020.6231

For peer review only

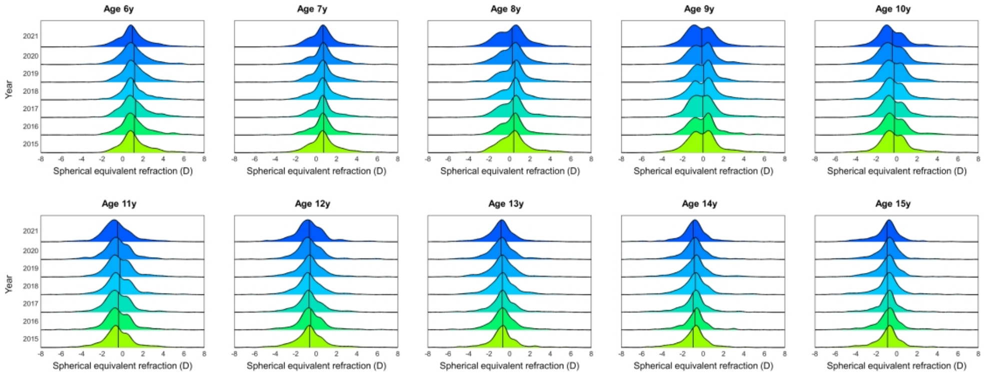

Figure 1. Spherical equivalent refraction (SER) distribution in children aged 6 to 15 years, between 2015 and 2021. SER distribution is plotted as a function of year and individual age groups. Within each distribution, the black vertical line indicates the mean. In 2020 and 2021, between 8 and 11 years of age, the vertical line is shifted towards more negative values. At other ages, the displacement of the vertical line is not so evident. Y-axis indicates the years, and x-axis represent SER in diopters.

464x175mm (300 x 300 DPI)

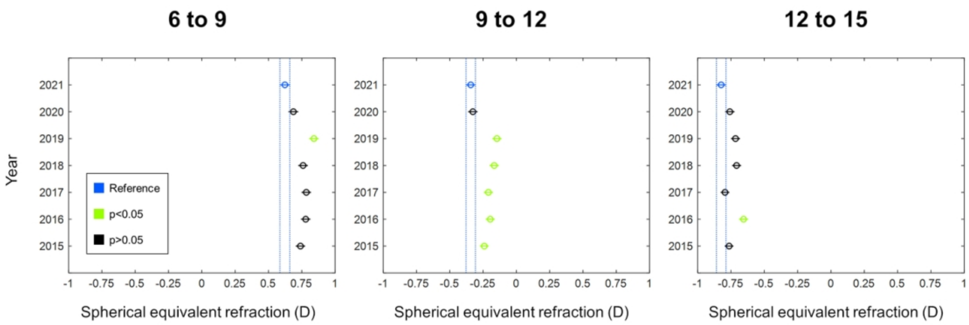

Figure 2. Mean spherical equivalent refraction (SER) in children clustered in three age groups (6 to 9, 9 to 12, and 12 to 15) between 2015 and 2021. Mean SER for 2021 was taken as a reference (in blue) and compared to previous years. Green circles reveal a comparison resulting in a P value less than 0.05, while black circles exhibit a P value greater than 0.05. In all three age ranges, 2021 shows the most negative SER values. The largest refractive differences between 2021 and previous years can be observed in the 9 to 12 age range. Y-axis displays the years, and the x-axis indicates SER in diopters. Error bars denote standard error of the mean.

124x41mm (300 x 300 DPI)

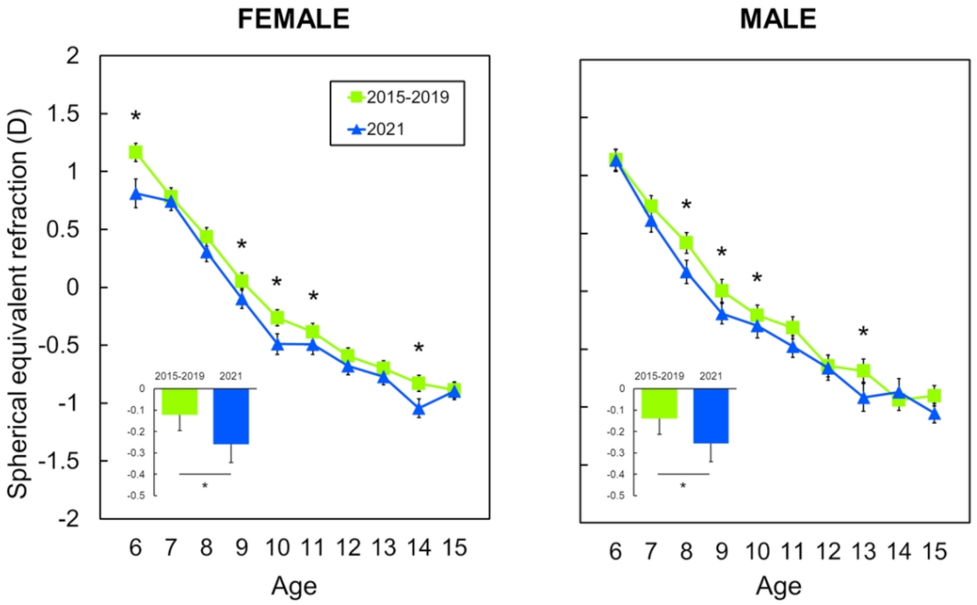

Figure 3. Mean spherical equivalent refraction (SER) values based on age and gender for 2015-2019 range and 2021. SER values for 2015-2019 range are provided as pooled values of the 5 consecutive years. SER values for 2015-2019 range are shown in green squares, while those for 2021 in blue triangles. Bar graph depicts mean SER values for 2015-2019 range and 2021 for all ages in both genders. Female group (left) and male group (right). Error bars represent standard error of the mean. Y-axis shows SER in diopters, and x-axis indicates age. Asterisks report statistical significance (p<0.05).

102x63mm (300 x 300 DPI)

STROBE 2007 (v4) Statement—Checklist of items that should be included in reports of *cross-sectional studies*

Section/Topic	Item #	Recommendation	Reported on page #
**Title and abstract**	1	(*a*) Indicate the study's design with a commonly used term in the title or the abstract	2, 3
		(*b*) Provide in the abstract an informative and balanced summary of what was done and what was found	3
**Introduction**			
Background/rationale	2	Explain the scientific background and rationale for the investigation being reported	5
Objectives	3	State specific objectives, including any prespecified hypotheses	5
**Methods**			
Study design	4	Present key elements of study design early in the paper	5-7
Setting	5	Describe the setting, locations, and relevant dates, including periods of recruitment, exposure, follow-up, and data collection	5-7
Participants	6	(*a*) Give the eligibility criteria, and the sources and methods of selection of participants	5-7
Variables	7	Clearly define all outcomes, exposures, predictors, potential confounders, and effect modifiers. Give diagnostic criteria, if applicable	5-7
Data sources/ measurement	8*	For each variable of interest, give sources of data and details of methods of assessment (measurement). Describe comparability of assessment methods if there is more than one group	5-7
Bias	9	Describe any efforts to address potential sources of bias	5-7
Study size	10	Explain how the study size was arrived at	5-7
Quantitative variables	11	Explain how quantitative variables were handled in the analyses. If applicable, describe which groupings were chosen and why	5-7
Statistical methods	12	(*a*) Describe all statistical methods, including those used to control for confounding	5-7
		(*b*) Describe any methods used to examine subgroups and interactions	5-7
		(*c*) Explain how missing data were addressed	5-7
		(*d*) If applicable, describe analytical methods taking account of sampling strategy	5-7
		(*e*) Describe any sensitivity analyses	5-7
**Results**			

For peer review only

Participants	13*	(a) Report numbers of individuals at each stage of study—eg numbers potentially eligible, examined for eligibility, confirmed eligible, included in the study, completing follow-up, and analysed	7-11
		(b) Give reasons for non-participation at each stage	7-11
		(c) Consider use of a flow diagram	7-11
Descriptive data	14*	(a) Give characteristics of study participants (eg demographic, clinical, social) and information on exposures and potential confounders	7-11
		(b) Indicate number of participants with missing data for each variable of interest	7-11
Outcome data	15*	Report numbers of outcome events or summary measures	7-11
Main results	16	(*a*) Give unadjusted estimates and, if applicable, confounder-adjusted estimates and their precision (eg, 95% confidence interval). Make clear which confounders were adjusted for and why they were included	7-11
		(*b*) Report category boundaries when continuous variables were categorized	7-11
		(*c*) If relevant, consider translating estimates of relative risk into absolute risk for a meaningful time period	7-11
Other analyses	17	Report other analyses done—eg analyses of subgroups and interactions, and sensitivity analyses	7-11
**Discussion**			
Key results	18	Summarise key results with reference to study objectives	12-15
Limitations	19	Discuss limitations of the study, taking into account sources of potential bias or imprecision. Discuss both direction and magnitude of any potential bias	15
Interpretation	20	Give a cautious overall interpretation of results considering objectives, limitations, multiplicity of analyses, results from similar studies, and other relevant evidence	12-15
Generalisability	21	Discuss the generalisability (external validity) of the study results	12-15
**Other information**			
Funding	22	Give the source of funding and the role of the funders for the present study and, if applicable, for the original study on which the present article is based	17

*Give information separately for cases and controls in case-control studies and, if applicable, for exposed and unexposed groups in cohort and cross-sectional studies.

Note: An Explanation and Elaboration article discusses each checklist item and gives methodological background and published examples of transparent reporting. The STROBE checklist is best used in conjunction with this article (freely available on the Web sites of PLoS Medicine at http://www.plosmedicine.org/, Annals of Internal Medicine at http://www.annals.org/, and Epidemiology at http://www.epidem.com/). Information on the STROBE Initiative is available at www.strobe-statement.org.

For peer review only

BMJ Open

Evaluating the impact of COVID-19 pandemic-related home confinement on the refractive error of school-aged children in Germany: a cross-sectional study based on data from 414 eye care professional centers

Journal:	*BMJ Open*
Manuscript ID	bmjopen-2023-071833.R1
Article Type:	Original research
Date Submitted by the Author:	21-Sep-2023
Complete List of Authors:	Sanz Diez, Pablo; Eberhard Karls Universitat Tubingen, Institute for Ophthalmic Research; Carl Zeiss Vision International GmbH, Technology and Innovation<br>Ohlendorf, Arne; Carl Zeiss Vision International GmbH, Technology and Innovation; Eberhard Karls Universitat Tubingen, Institute for Ophthalmic Research<br>Barraza-Bernal, Maria Jose; Carl Zeiss Vision GmbH, Technology and Innovation<br>Kratzer, Timo; Carl Zeiss Vision GmbH, Technology and Innovation<br>Wahl, Siegfried; Carl Zeiss Vision International GmbH, Technology and Innovation; Eberhard Karls Universitat Tubingen, Institute for Ophthalmic Research
**Primary Subject Heading**:	Ophthalmology
Secondary Subject Heading:	Ophthalmology, Paediatrics, Public health
Keywords:	COVID-19, Public health < INFECTIOUS DISEASES, Paediatric ophthalmology < OPHTHALMOLOGY

SCHOLARONE™
Manuscripts

I, the Submitting Author has the right to grant and does grant on behalf of all authors of the Work (as defined in the below author licence), an exclusive licence and/or a non-exclusive licence for contributions from authors who are: i) UK Crown employees; ii) where BMJ has agreed a CC-BY licence shall apply, and/or iii) in accordance with the terms applicable for US Federal Government officers or employees acting as part of their official duties; on a worldwide, perpetual, irrevocable, royalty-free basis to BMJ Publishing Group Ltd ("BMJ") its licensees and where the relevant Journal is co-owned by BMJ to the co-owners of the Journal, to publish the Work in this journal and any other BMJ products and to exploit all rights, as set out in our licence.

The Submitting Author accepts and understands that any supply made under these terms is made by BMJ to the Submitting Author unless you are acting as an employee on behalf of your employer or a postgraduate student of an affiliated institution which is paying any applicable article publishing charge ("APC") for Open Access articles. Where the Submitting Author wishes to make the Work available on an Open Access basis (and intends to pay the relevant APC), the terms of reuse of such Open Access shall be governed by a Creative Commons licence – details of these licences and which Creative Commons licence will apply to this Work are set out in our licence referred to above.

Other than as permitted in any relevant BMJ Author's Self Archiving Policies, I confirm this Work has not been accepted for publication elsewhere, is not being considered for publication elsewhere and does not duplicate material already published. I confirm all authors consent to publication of this Work and authorise the granting of this licence.

For peer review only

Evaluating the impact of COVID-19 pandemic-related home confinement on the refractive error of school-aged children in Germany: a cross-sectional study based on data from 414 eye care professional centers

Pablo Sanz Diez, PhD [1, 3 *]; Arne Ohlendorf, PhD [1, 3]; Maria Barraza-Bernal, PhD [2]; Timo Kratzer [2]; Siegfried Wahl, Prof. [1,3]

1. Carl Zeiss Vision International GmbH, Technology & Innovation, Aalen, Germany.
2. Carl Zeiss Vision GmbH, Technology & Innovation, Aalen, Germany.
3. Institute for Ophthalmic Research, Eberhard Karls University Tuebingen, Tuebingen, Germany.

Corresponding author's contact information:

Pablo Sanz Diez

Carl Zeiss Vision International GmbH, Technology & Innovation

Institute for Ophthalmic Research, Eberhard Karls University Tuebingen

Elfriede-Aulhorn-Strasse 7

72076 Tuebingen, Germany

Phone: + 49 7071 29 80810

pablo.sanzdiez@zeiss.com

For peer review only

Abstract

Objective: This study aimed at evaluating refractive changes in German school-aged children before and after the COVID-19 pandemic.

Design: Cross-sectional study.

Setting: 414 eye care professional centers from Germany.

Participants: Refractive data from 59926 German children aged 6 to 15 years were examined over a 7-year period (2015-2021).

Primary and secondary outcome measures: Spherical equivalent refraction was assessed as a function of year, age, and gender. The refractive values concerning 2020 and 2021 were compared with those assigned to prior years (2015-2019).

Results: The refractive data associated with 2020 and 2021 showed a myopic refractive shift of approximately -0.20D compared to the 2015-2019 range. The refractive change was statistically considerable in the 6- to 11-year range ($p<0.05$), while from 12 to 15 years was negligible ($p\geq0.10$). Percentage of myopes was also impacted in 2021 ($p=0.002$), but not in 2020 ($p=0.25$). From 6 to 11 years, the percentage of myopes in 2021 increased significantly by 6.02% compared to the 2015–2019 range ($p\leq0.04$). The highest percentage increase occurred at 8 and 10 years of age, showing a rise of 7.42% ($p=0.002$) and 6.62% ($p=0.005$), respectively. From 12 to 15 years, there was no significant increase in the percentage of myopes in 2021 ($p\geq0.09$). Percentage of myopes in 2020 was not influenced at any age ($p\geq0.06$).

Conclusion: Disruption of normal lifestyle due to pandemic-related home confinement appears to lead to a myopic refractive shift in children aged 6 to 11 years in Germany. The greater effect observed at younger ages seems to emphasize the importance of refractive development in this age group.

For peer review only

Strengths and limitations of this study

- This is the first study aimed at reporting the impact of COVID-19 pandemic-related home confinement on the refractive error of school-aged children in Germany.
- Data were collected from a network management software from a total of 414 eye care professional centers in Germany. Data from such a diverse set of centers increases the representativeness of the sample and the generalizability of study's findings making them more applicable to a wider population.
- Refraction data analysis of 7-year period, from 2015-2021. The current study is one of the few studies that includes data from 2021 to explore the effect of COVID-19 pandemic-related home confinement on the refractive error of school-aged children. Such long-term data provide valuable insights into how home confinement during the pandemic may have affected the refractive error of these children over time, as well as identifying any potential trends or patterns.
- The nature of the database collected by a network management software for eye care professional centers may limit additional information that could help to better understand and interpret the obtained results.

For peer review only

Introduction

Coronavirus disease 2019 (COVID-19), characterized as a pandemic by the World Health Organization on March 11, 2020 [1], has resulted in a global health, social, and economic crisis [2, 3]. Containment and control of the disease was the priority strategy adopted by most governments. Mandatory use of masks, social distancing, self-isolation, and nationwide home confinement were the main measures aimed at curbing the spread of the disease [4]. In many countries, the home confinement policy has restricted citizens from leaving their homes except for justified reasons, which has led to many negative psychosocial and psychological consequences [5-7]. Regarding ocular health, the increased time spent indoors and working on near work activities, such as screen time, reading, writing, among many others, both linked to myopia development [8, 9], has raised interest about the impact of home confinement on the refractive status of school-aged children. In fact, changes in the mean spherical equivalent refraction and increased myopia prevalence have already been clearly reported as collateral consequences in school-aged children [10-17]. The purpose of the current study was to assess whether COVID-19 pandemic-related home confinement caused refractive changes in school-aged children in Germany.

For peer review only

Methods

Study dataset

The dataset used for the analyses described in the current study were obtained from Euronet (Euronet Market Research, Euronet Software AG, Frechen, Germany). Euronet is a network management software for eye care centers, which has been administering clinical and ocular history data in Germany since 2001.

For the current study, the dataset was comprised of the following variables: center identification number, subject identification number, purchase date, date of birth, age, gender, spectacle-plane refractive correction (sphere, cylinder, axis), and visual acuity.

Study population

First-visit refractive data from 67137 children aged 6 to 15 years were collected from 414 eye care professional (ECP) centers in Germany between 2015 and 2021.

For the final analysis, 34300 females and 25626 males were considered. A total of 7211 cases were excluded due to incomplete gender information.

The present study was in accordance with the declaration of Helsinki. All data involved in the current research were de-identified and protected by the privacy safeguards of the European General Data Protection Regulation.

Statistical Analysis

Statistical analyses were conducted under the MATLAB R2020a statistics and machine learning toolbox (MathWorks, Massachusetts, USA). Statistical tests were chosen according to the study purposes and data distribution, which was previously assessed by means of the Kolmogorov-Smirnov test. Differences in the yearly refractive error were examined by two-sided Mann–Whitney U test or Kruskal-Wallis test followed by Tukey-Kramer post-hoc test. A two-proportion Z-test was used to determine statistical differences in the percentage of myopes among the evaluated years. Differences were considered statistically significant when $p<0.05$.

All values shown here represent spherical equivalent refraction (SER) data. SER values were calculated as the sum of the sphere power with half of the negative cylinder power. The purchase date was the variable considered to establish the SER values as a function of year. Only the data from the right eye was used for the final analysis. For the percentage of myopes assessment, myopia was defined as SER ≤ -0.50D.

For peer review only

Patient and public involvement

None of the individuals were involved in the design, implementation, reporting or dissemination plans of our research.

Results

Data from a total of 59926 German children aged 6 to 15 years were evaluated in this study. 34300 females (57.24%; mean age: 10.82±2.84 years; and mean SER: -0.22±1.79D) and 25626 males (42.76%; mean age: 10.40±2.88 years; and mean SER: -0.15±1.93D) comprised the reported outcomes. Among the two gender groups, the mean SER values was -0.19±1.85D, while the minimum and maximum SER values were -24.25 and 17.50D, respectively. The most positive mean SER was found in the 6-year age group (1.12±1.78D), while the most negative mean SER among children by age of 15 (-0.91±1.68D). On average, SER was age-dependent (χ^2=6250.63, $p<0.001$; and χ^2=4676.7, $p<0.001$, for females and males respectively), and gender-dependent for specific age groups. From 6 to 11 years, both gender groups showed similar SER values ($p\geq0.07$), whereas from 12 to 15 years, males exhibited more negative SER values compared to females in their respective age groups ($p<0.02$).

Figure 1 and supplemental table 1 show the SER distribution and mean SER values for individual age groups along a 7-year period (2015-2021). On average, among all years, 2020 and 2021 presented the highest negative SER values, being 2021 the most statistically evident (supplemental table 1). From 2015 to 2019, mean SER values remain stable towards more positive readings. A comparison of 2020 and 2021 with previous years revealed a 0.2D myopic shift in those children aged 6 to 11 years ($p<0.05$). Between 12 to 15 years, the myopic shift in 2020 and 2021 was less than 0.08D ($p\geq0.10$).

For peer review only

Analyzing the data by three age ranges evidenced 2021 as the year with the highest myopic mean SER values (Figure 2). As seen in Figure 2, considering 2021 as benchmark revealed maximum SER differences of -0.22D (95% CI: -0.37 to -0.07; p<0.001), -0.20D (95% CI: -0.34 to -0.05; p<0.001) and -0.17D (95% CI: -0.31 to -0.02; p<0.001) in the age ranges 6 to 9, 9 to 12, and 12 to 15, respectively. 9 to 12 age range showed statistically significant differences among 2021 and all previous years, from 2015 to 2019 (χ^2=49.41, p<0.001; post-hoc: p<0.004 all). Smaller SER differences were found by comparing 2020 and prior years (Figure 2). Here, 2020 exhibited maximum SER differences of -0.15D (95% CI: -0.31 to 0.00; p=0.04), -0.18D (95% CI: -0.33 to -0.04; p<0.01) and -0.10D (95% CI: -0.25 to 0.04; p<0.01) for 6 to 9, 9 to 12, and 12 to 15 age ranges, respectively.

The myopic shift observed in 2021 seems to be followed by an increase in the percentage of myopes in children aged 6 to 11 years (supplemental table 2). On average, in this age range, the percentage of myopes in 2021 increased significantly by 6.02% compared to the 2015–2019-year range (p≤0.04; Z≤.49). The highest increase in the percentage of myopes was found at 8 and 10 years of age, showing a rise of 7.42% (p=0.002; Z=-3.17) and 6.62% (p=0.005; Z=-2.78), respectively. From 12 to 15 years, no significant changes in the percentage of myopes in 2021 were found (p≥0.09; Z≥-1.72). Instead, as seen in supplemental table 2, no significant changes in the percentage of myopes at any age were noted in 2020 relative to the 2015-2019 range (p=0.25; Z=-1.16 and p≥0.06; Z≥-1.91 for all ages).

Gender-specific effects were depicted in Figure 3. Sample sizes by age, gender and year are presented in supplemental table 3. When comparing 2021 with the 2015-2019 range, both genders showed similar refractive trends. The largest refractive differences occurred between 6 and 11 years of age (Figure 3). Here, 2021 showed a mean refractive shift of -0.17D and -0.16D for females and males, respectively. Within the range 2015-2019, 2019 was the year with the biggest refractive differences relative to 2021. Females experienced the greatest refractive shift from 6 to 11 years (-0.27D), while for males it was among 8 and 13 years (-0.21D).

For peer review only

Discussion

In the present study we have shown refractive data concerning a period of 7 consecutive years (2015-2021) in a population of children aged 6 to 15 years in Germany. In the 2015-2019 range, the mean SER values showed stable tendencies with no meaningful variations among them. However, in 2020 and 2021, we found a shift in SER toward more myopic values when compared to previous years (Figure 1, Figure 2, and supplemental table 1). Interestingly, the refractive shift appeared to be dependent on the population age. From 6 to 11 years, children showed a significant mean refractive shift of approximately -0.20D, while from 12 to 15 years, the mean change was smaller than -0.08D.

Examining the 2020 and 2021 refractive data, 2021 was revealed as the year with the most prominent shift. All these refractive trends were observed in both males and females, with the 6- to 11-year-old range again being the most influenced.

Percentage of myopes was also affected, however, only in 2021. Again, the statistically significant changes were noted in the 6- to 11-year range. Here, the percentage of myopes in 2021 was on average 6% higher than in the 2015-2019 range. In contrast, no significant changes in the percentage of myopes were observed in 2020. These findings reinforce 2021 as the most impacted year.

Our findings agree with those studies reporting the impact of COVID-19 home confinement on myopia development in schoolchildren [10-17]. Wang J et al. [10], from 2015 to 2020, measured the refractive data of 123535 children (59200 females and 64335 males) aged 6 to 13 years from ten primary schools in Feicheng, China. Authors found that children aged 6 to 8 years exhibited a mean myopic shift of -0.30D, while those aged 9 to 13 years less than -0.10D. A 1.4 to 3-fold increase in myopia prevalence was also noted in children aged 6 to 8 years (15.8% for 6 years, 10% for 7 years, and 9.5% for 8 years). No significant increase was detected in 9- to 13-year-olds [10]. Other Asian population-based studies revealed similar results. Xu L et al. [12]

For peer review only

observed a mean increase in myopia prevalence of 6.50%, which was school-grade dependent, 8.54% (from grades 1 to 6) and 4.32% (from grades 7 to 12). Myopia progression increased 1.5 times and was faster in the youngest, grades 1 to 6 [12]. Hu Y et al. [11] found that grade 3 students experienced a myopic SER shift of -0.35D and a myopia prevalence increase of 7.5%. Ma D et al. [17] found a -0.6D change in SER in children aged 8 to 10 years following 7-month period of home study during the pandemic. A similar refractive change was reported by Ma M et al. [16] after a refractive screening of 201 myopic children aged 7 to 12 years during the period from April 2019 to May 2020. In May 2020, a refractive shift of -0.59D was determined [16]. Yang X et al. [15] found a myopic SER change but only in low hyperopia, emmetropia, and mild myopia in school grades 1-4 and 7. On average, between 2019 and 2020, the reported SER change was -0.39D [15].

In Europe, few studies have evaluated the effect of pandemic home confinement on children's refractive development. A cross-sectional study in Spanish children aged 5 to 7 years reported a mean SER shift of -0.18 in 2020 compared with 2019. In their study population, the myopia percentage remained stable, while hyperopia and emmetropia decreased and increased, respectively [14]. Likewise, in 2021, Italian children aged 5 to 12 years showed a mean SER reduction of -0.50D and a mean increase in myopia prevalence of 9.12%. Those aged 9 to 12 years were the most influenced [13].

These findings, in both Asian and European populations, are consistent with ours. Overall, after the pandemic home confinement period, we have observed a SER myopic shift and an increase in the percentage of myopes in the studied German school-aged population. As described in prior studies [10, 12, 13, 15], the refractive aftereffects established in our study were also age dependent. While no effect was seen from 12 to 15 years of age, the most important refractive changes occurred in the age range of 6 to 11 years. Specifically, the highest refractive and myopia percentage changes appeared at ages 8 to 11 years, compatible with the age range described in Asian [10, 12, 15] and Italian [13] children. As postulated by Wang et al. [10],

For peer review only

younger children seem to be more sensitive to environmental changes than older ones. This hypothesis is supported by those studies that have already documented a faster myopia progression [18] and axial elongation [19] at younger ages. Apparently, the ocular and refractive plasticity involved at this age window may be crucial [10], however further assessments are required to draw more robust assumptions.

As previously seen, the refractive and percentage change rates calculated in the current study were comparable but not strictly equal to those reported by other authors. Small refractive variations among studies may be due to multiple reasons: application of different refraction techniques, genetic predisposition of the study population, interaction of environmental factors or even different home confinement regulations. Perhaps the more restrictive nationwide confinement regulations established in China or Italy, compared to Germany, have made the refractive aftereffects even higher and already visible in 2020, as seen in the Asian population-based studies. As previously reported, in our study the refractive aftereffects were found to be more evident in 2021 rather than in 2020. The effects of certain myopia risk factors related to the pandemic, such as increased screen time, reduced outdoor time, disruptions to healthcare access, as well as increased stress and anxiety may take time to appear or to be detected in terms of myopia. For example, increased screen time and reduced outdoor time, due to COVID-19 restrictions, may not immediately lead to myopia, but may contribute to its development over time. Changes in healthcare access may also take time to impact myopia prevalence, as delays in diagnosis and treatment may not be immediately apparent. Stress and anxiety may not cause myopia directly but may exacerbate existing myopia or make it more difficult to manage. More research is needed to fully understand the time course of these effects and to identify any other potential contributing factors. It is also important to consider the impact of individual differences and environmental factors on the relationship between the COVID-19 pandemic and myopia. Additionally, it should be noted that most of the published studies on the refractive effects of

For peer review only

COVID-19 pandemic-related home confinement did not analyze refractive data from 2021. It would be interesting to see if they also find a greater refractive effect in 2021 compared to 2020.

Undeniably, lifestyle behavioral changes induced by the COVID-19 pandemic have led to refractive changes in school-aged children. Government regulations such as home confinement have mainly resulted in increased near work activities, and reduced time spent outdoors, both risk factors for myopia [8, 9]. All these results should warn governmental authorities when planning any nationwide lockdown with home confinement [20]. Further refractive studies over the next few years are needed to reveal whether this is a reversible outcome or not.

Limitations

The database used in the current study is sourced from a network management software for eye care centers. This database is anonymous and lacks certain information such as the type of refraction techniques used or the individual's ocular history data. Consequently, these inherent characteristics give rise to certain study limitations that need to be addressed.

On one side, we cannot ascertain the specific refractive techniques employed to obtain the refractive data. This aspect could introduce measurement inaccuracies that may impact our findings. However, we have implemented statistical methodologies that exhibit robustness against possible inaccuracies. Additionally, the absence of comprehensive data related to ocular history could potentially serve as a substantial limitation in our research. This insufficiency may inadvertently result in the inclusion of subjects with specific ocular pathologies that would ordinarily be considered exclusion criteria.

On the other side, our database lacked ocular biometric data such as axial length, anterior chamber depth, corneal curvature, and lens thickness. Moreover, the absence of lifestyle data,

For peer review only

such as time spent outdoors and time in near work activities, further restricted our ability to comprehensively analyze the potential factors influencing refractive changes.

Despite these limitations, it is important to acknowledge that the database also offers several advantages. Its extensive size provides a substantial increase in the statistical power of the study, enhancing the robustness of our analysis. Moreover, the diverse sources of data from various eye care professional centers enhance the sample's representativeness and broaden the generalizability of our study findings, making them more applicable to a wider population.

Overall, while we recognize the inherent limitations of our data source, we believe that our outcomes agree with prior research and may offer valuable insights into the impact of COVID-19 pandemic-related home confinement on the refractive error of school-aged children in Germany. Nevertheless, it is crucial to approach these findings with caution, and therefore, additional research would be necessary to uncover the potential causal mechanisms that may underlie our observations.

Conclusion

Our study provides preliminary insights into the impact of COVID-19 pandemic-related home confinement on the refractive error of school-aged children in Germany. In summary our findings suggest a potential association between home confinement and a myopic refractive shift in a subset of German children aged 6 to 11 years, according to the 2020 and 2021 refractive data. Interestingly, within this cohort, a more prominent trend emerges among children aged 8 to 11, aligning with the importance of this age range during myopia development. Nonetheless, this observation underscores the need for in-depth exploration and further investigations.

For peer review only

Contributors: PSD, AO, MBB, TK, and SW conceived and planned the study. PSD, AO, and MBB performed the data analysis and data interpretation. TK and SW served as project manager for the study. PSD wrote the first draft of the manuscript. PSD AO, MBB, TK, and SW conducted the subsequent revisions. PSD, AO, MBB, TK, and SW provided technical and intellectual aspects for the study.

Competing interests: All authors declare no competing interests.

Funding: This research received no specific grant from any funding agency in the public, commercial or not-for-profit sectors.

Data sharing statement: Data are available upon reasonable request.

Ethics approval statement: The present study was in accordance with the declaration of Helsinki. All data involved in the current research were de-identified and protected by the privacy safeguards of the European General Data Protection Regulation.

For peer review only

References

[1] World Health Organization., "WHO Director-General's opening remarks at the media briefing on COVID-19-11 March 2020," ed. Geneva, Switzerland 2020.

[2] C. A. Pollard, M. P. Morran, and A. L. Nestor-Kalinoski, "The COVID-19 pandemic: a global health crisis," *Physiol Genomics,* vol. 52, no. 11, pp. 549-557, Nov 1 2020, doi: 10.1152/physiolgenomics.00089.2020.

[3] M. Nicola *et al.*, "The socio-economic implications of the coronavirus pandemic (COVID-19): A review," *Int J Surg,* vol. 78, pp. 185-193, Jun 2020, doi: 10.1016/j.ijsu.2020.04.018.

[4] K. Farsalinos *et al.*, "Improved strategies to counter the COVID-19 pandemic: Lockdowns vs. primary and community healthcare," *Toxicol Rep,* vol. 8, pp. 1-9, 2021, doi: 10.1016/j.toxrep.2020.12.001.

[5] S. Rohr, F. Muller, F. Jung, C. Apfelbacher, A. Seidler, and S. G. Riedel-Heller, "[Psychosocial Impact of Quarantine Measures During Serious Coronavirus Outbreaks: A Rapid Review]," *Psychiatr Prax,* vol. 47, no. 4, pp. 179-189, May 2020, doi: 10.1055/a-1159-5562. Psychosoziale Folgen von Quarantanemassnahmen bei schwerwiegenden Coronavirus-Ausbruchen: ein Rapid Review.

[6] S. K. Brooks *et al.*, "The psychological impact of quarantine and how to reduce it: rapid review of the evidence," *Lancet,* vol. 395, no. 10227, pp. 912-920, Mar 14 2020, doi: 10.1016/S0140-6736(20)30460-8.

[7] K. F. Ahrens *et al.*, "Impact of COVID-19 lockdown on mental health in Germany: longitudinal observation of different mental health trajectories and protective factors," *Transl Psychiatry,* vol. 11, no. 1, p. 392, Jul 17 2021, doi: 10.1038/s41398-021-01508-2.

[8] J. C. Sherwin, M. H. Reacher, R. H. Keogh, A. P. Khawaja, D. A. Mackey, and P. J. Foster, "The association between time spent outdoors and myopia in children and adolescents: a systematic review and meta-analysis," *Ophthalmology,* vol. 119, no. 10, pp. 2141-51, Oct 2012, doi: 10.1016/j.ophtha.2012.04.020.

[9] M. He *et al.*, "Effect of Time Spent Outdoors at School on the Development of Myopia Among Children in China: A Randomized Clinical Trial," *JAMA,* vol. 314, no. 11, pp. 1142-8, Sep 15 2015, doi: 10.1001/jama.2015.10803.

[10] J. Wang *et al.*, "Progression of Myopia in School-Aged Children After COVID-19 Home Confinement," *JAMA Ophthalmol,* vol. 139, no. 3, pp. 293-300, Mar 1 2021, doi: 10.1001/jamaophthalmol.2020.6239.

[11] Y. Hu *et al.*, "Rates of Myopia Development in Young Chinese Schoolchildren During the Outbreak of COVID-19," *JAMA Ophthalmol,* vol. 139, no. 10, pp. 1115-1121, Oct 1 2021, doi: 10.1001/jamaophthalmol.2021.3563.

[12] L. Xu *et al.*, "COVID-19 Quarantine Reveals That Behavioral Changes Have an Effect on Myopia Progression," *Ophthalmology,* vol. 128, no. 11, pp. 1652-1654, Nov 2021, doi: 10.1016/j.ophtha.2021.04.001.

[13] E. Trovato Battagliola *et al.*, "Effect of COVID-19 lockdown on refractive errors in Italian children aged 5-12 years: A multi-center retrospective study," *Eur J Ophthalmol,* p. 11206721221106135, Jun 9 2022, doi: 10.1177/11206721221106135.

[14] C. Alvarez-Peregrina, C. Martinez-Perez, C. Villa-Collar, C. Andreu-Vazquez, A. Ruiz-Pomeda, and M. A. Sanchez-Tena, "Impact of COVID-19 Home Confinement in Children's Refractive Errors," *Int J Environ Res Public Health,* vol. 18, no. 10, May 17 2021, doi: 10.3390/ijerph18105347.

[15] X. Yang *et al.*, "Changes in Refractive Error Under COVID-19: A 3-Year Follow-up Study," *Adv Ther,* vol. 39, no. 6, pp. 2999-3010, Jun 2022, doi: 10.1007/s12325-022-02150-0.

For peer review only

[16] M. Ma, S. Xiong, S. Zhao, Z. Zheng, T. Sun, and C. Li, "COVID-19 Home Quarantine Accelerated the Progression of Myopia in Children Aged 7 to 12 Years in China," *Invest Ophthalmol Vis Sci,* vol. 62, no. 10, p. 37, Aug 2 2021, doi: 10.1167/iovs.62.10.37.

[17] D. Ma *et al.*, "Progression of myopia in a natural cohort of Chinese children during COVID-19 pandemic," *Graefes Arch Clin Exp Ophthalmol,* vol. 259, no. 9, pp. 2813-2820, Sep 2021, doi: 10.1007/s00417-021-05305-x.

[18] L. A. Jones-Jordan *et al.*, "Myopia Progression as a Function of Sex, Age, and Ethnicity," *Invest Ophthalmol Vis Sci,* vol. 62, no. 10, p. 36, Aug 2 2021, doi: 10.1167/iovs.62.10.36.

[19] P. Sanz Diez, L. H. Yang, M. X. Lu, S. Wahl, and A. Ohlendorf, "Growth curves of myopia-related parameters to clinically monitor the refractive development in Chinese schoolchildren," *Graefes Arch Clin Exp Ophthalmol,* vol. 257, no. 5, pp. 1045-1053, May 2019, doi: 10.1007/s00417-019-04290-6.

[20] C. C. W. Klaver, J. R. Polling, and C. A. Enthoven, "2020 as the Year of Quarantine Myopia," *JAMA Ophthalmol,* vol. 139, no. 3, pp. 300-301, Mar 1 2021, doi: 10.1001/jamaophthalmol.2020.6231.

For peer review only

Figure legends

Figure 1. Spherical equivalent refraction (SER) distribution in children aged 6 to 15 years, between 2015 and 2021. SER distribution is plotted as a function of year and individual age groups. Within each distribution, the black vertical line indicates the mean. In 2020 and 2021, between 8 and 11 years of age, the vertical line is shifted towards more negative values. At other ages, the displacement of the vertical line is not so evident. Y-axis indicates the years, and x-axis represent SER in diopters.

Figure 2. Mean spherical equivalent refraction (SER) in children clustered in three age groups (6 to 9, 9 to 12, and 12 to 15) between 2015 and 2021. Mean SER for 2021 was taken as a reference (in blue) and compared to previous years. Green circles reveal a comparison resulting in a P value less than 0.05, while black circles exhibit a P value greater than 0.05. In all three age ranges, 2021 shows the most negative SER values. The largest refractive differences between 2021 and previous years can be observed in the 9 to 12 age range. Y-axis displays the years, and the x-axis indicates SER in diopters. Error bars denote standard error of the mean.

Figure 3. Mean spherical equivalent refraction (SER) values based on age and gender for 2015-2019 range and 2021. SER values for 2015-2019 range are provided as pooled values of the 5 consecutive years. SER values for 2015-2019 range are shown in green squares, while those for 2021 in blue triangles. Bar graph depicts mean SER values for 2015-2019 range and 2021 for all ages in both genders. Female group (left) and male group (right). Error bars represent standard error of the mean. Y-axis shows SER in diopters, and x-axis indicates age. Asterisks report statistical significance ($p<0.05$).

For peer review only

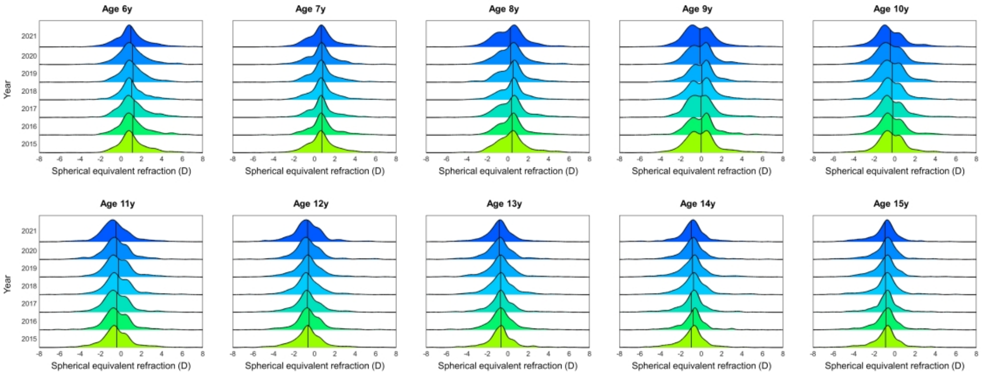

Figure 1. Spherical equivalent refraction (SER) distribution in children aged 6 to 15 years, between 2015 and 2021. SER distribution is plotted as a function of year and individual age groups. Within each distribution, the black vertical line indicates the mean. In 2020 and 2021, between 8 and 11 years of age, the vertical line is shifted towards more negative values. At other ages, the displacement of the vertical line is not so evident. Y-axis indicates the years, and x-axis represent SER in diopters.

464x175mm (394 x 394 DPI)

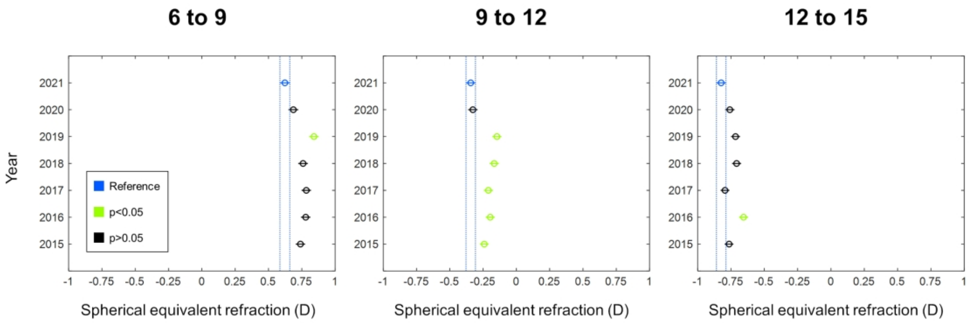

Figure 2. Mean spherical equivalent refraction (SER) in children clustered in three age groups (6 to 9, 9 to 12, and 12 to 15) between 2015 and 2021. Mean SER for 2021 was taken as a reference (in blue) and compared to previous years. Green circles reveal a comparison resulting in a P value less than 0.05, while black circles exhibit a P value greater than 0.05. In all three age ranges, 2021 shows the most negative SER values. The largest refractive differences between 2021 and previous years can be observed in the 9 to 12 age range. Y-axis displays the years, and the x-axis indicates SER in diopters. Error bars denote standard error of the mean.

124x41mm (600 x 600 DPI)

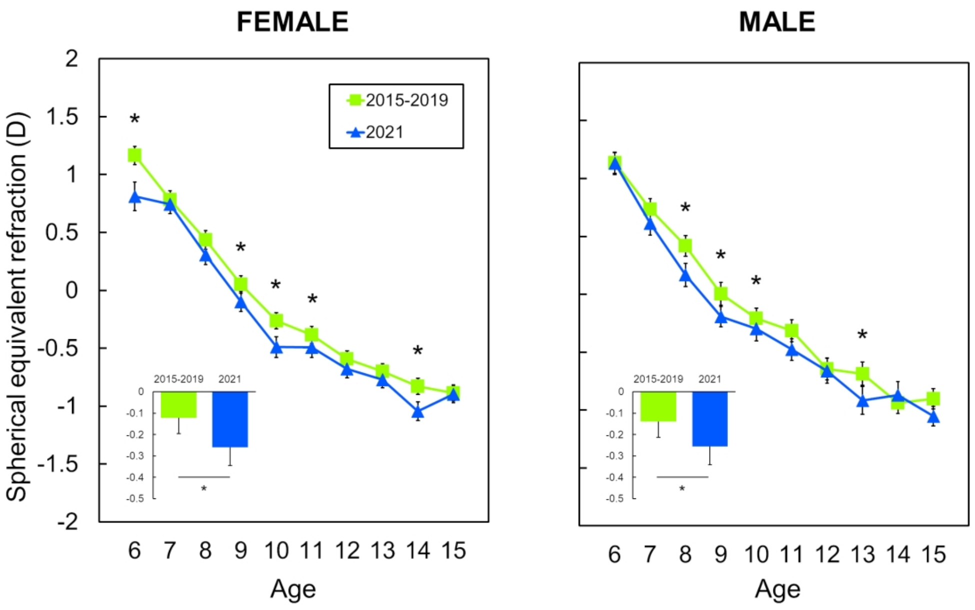

Figure 3. Mean spherical equivalent refraction (SER) values based on age and gender for 2015-2019 range and 2021. SER values for 2015-2019 range are provided as pooled values of the 5 consecutive years. SER values for 2015-2019 range are shown in green squares, while those for 2021 in blue triangles. Bar graph depicts mean SER values for 2015-2019 range and 2021 for all ages in both genders. Female group (left) and male group (right). Error bars represent standard error of the mean. Y-axis shows SER in diopters, and x-axis indicates age. Asterisks report statistical significance (p<0.05).

102x63mm (600 x 600 DPI)

Supplemental table 1. Mean annual spherical equivalent refraction (SER) values based on individual age groups.

Age (years)	Sample size	2015	2016	2017	2018	2019	2020	2021	p value (2020) [a]	p value (2021) [b]
**6**	5579	1.11(0.06)	1.16(0.06)	1.27(0.07)	1.04(0.06)	1.18(0.05)	1.12(0.07)	0.97(0.08)	0.80	0.03
**7**	5540	0.73(0.06)	0.75(0.06)	0.74(0.06)	0.79(0.05)	0.81(0.06)	0.71(0.06)	0.68(0.07)	0.96	0.32
**8**	5700	0.41(0.06)	0.42(0.06)	0.37(0.06)	0.45(0.06)	0.51(0.06)	0.27(0.06)	0.24(0.07)	0.09	0.005
**9**	5779	-0.01(0.06)	0.03(0.06)	-0.02(0.06)	0.10(0.06)	0.06(0.06)	-0.12(0.06)	-0.14(0.06)	0.11	<0.001
**10**	6272	-0.27(0.05)	-0.22(0.05)	-0.19(0.06)	-0.28(0.05)	-0.23(0.05)	-0.43(0.06)	-0.41(0.07)	0.001	<0.001
**11**	6009	-0.43(0.06)	-0.38(0.06)	-0.42(0.06)	-0.30(0.06)	-0.25(0.06)	-0.43(0.06)	-0.49(0.06)	0.44	<0.001
**12**	6068	-0.64(0.06)	-0.52(0.06)	-0.69(0.06)	-0.65(0.06)	-0.57(0.06)	-0.58(0.06)	-0.67(0.06)	0.64	0.29
**13**	6192	-0.67(0.05)	-0.66(0.06)	-0.73(0.06)	-0.71(0.06)	-0.72(0.06)	-0.83(0.06)	-0.83(0.06)	0.01	0.01
**14**	6262	-0.98(0.05)	-0.79(0.06)	-0.97(0.06)	-0.76(0.06)	-0.86(0.06)	-0.86(0.06)	-0.97(0.07)	0.42	0.18
**15**	6525	-0.89(0.05)	-0.86(0.06)	-0.86(0.05)	-0.90(0.05)	-0.96(0.06)	-0.93(0.06)	-0.95(0.06)	0.83	0.19

For each year, the values shown are mean SER and standard error of the mean. Between parentheses, the standard error of the mean. Values are given in diopters.

[a] p value associated with the comparison between SER values in 2020 and the averaged SER values among 2015-2019 for each age.

[b] p value associated with the comparison between SER values in 2021 and the averaged SER values among 2015-2019 for each age.

For peer review only

Supplemental table 2. Percentage of myopes as a function of age (6-15) and year (2015-2021).

Age (years)	Sample size	2015	2016	2017	2018	2019	2020	2021	p value (2020) [a]	Z value (2020) [b]	p value (2021) [c]	Z value (2021) [d]	p value (2020) [e]	Z value (2020) [f]	p value (2021) [g]	Z value (2021) [h]	p value (2020) [i]	Z value (2020) [j]	p value (2021) [k]	Z value (2021) [l]
**6**	5579	10.92	12.27 [m]	12.18	9.97	10.51	12.16	16.09	0.94	0.07	0.03	-2.19	0.54	-0.61	0.005	-2.83	0.25	-1.16	0.002	-3.15
**7**	5540	20.67 [m]	18.10	18.82	17.68	18.35	20.47	21.07	0.92	0.10	0.84	-0.20	0.39	-0.87	0.25	-1.16				
**8**	5700	29.44	27.88	30.90 [m]	26.65	27.17	32.00	35.82	0.62	-0.49	0.03	-2.12	0.11	-1.59	0.002	-3.17				
**9**	5779	43.72 [m]	40.94	43.52	42.17	38.46	44.35	48.09	0.80	-0.26	0.07	-1.80	0.30	-1.06	0.01	-2.58				
**10**	6272	50.85	50.67	51.50 [m]	50.67	49.43	54.67	57.25	0.19	-1.31	0.02	-2.40	0.09	-1.68	0.005	-2.78				
**11**	6009	56.32	53.96	56.41 [m]	52.20	52.17	53.02	59.03	0.17	1.39	0.29	-1.07	0.62	0.49	0.04	-1.98				
**12**	6068	61.15	58.55	63.07	63.32 [m]	59.18	61.49	60.48	0.44	0.77	0.24	1.17	0.86	-0.18	0.81	0.24				
**13**	6192	65.40 [m]	61.91	63.41	63.53	62.93	67.76	67.43	0.29	-1.06	0.37	-0.89	0.06	-1.91	0.09	-1.72				
**14**	6262	70.81 [m]	66.74	68.88	66.25	67.91	69.07	69.45	0.43	0.79	0.54	0..62	0.67	-0.42	0.55	-0.60				
**15**	6525	67.98	67.62	69.92	68.42	70.31 [m]	66.86	70.22	0.12	1.56	0.97	0.04	0.36	0.91	0.53	-0.63				

Myopia is defined as SER ≤ −0.50D. Values are expressed in percent. P values are calculated based on two proportion Z-test.

[a] p value associated with the comparison between the percentage of myopes in 2020 and the highest percentage in 2015-2019.

[b] Z statistic value associated with the comparison between the percentage of myopes in 2020 and the highest percentage in 2015-2019.

[c] p value associated with the comparison between the percentage of myopes in 2021 and the highest percentage in 2015-2019.

[d] Z statistic value associated with the comparison between the percentage of myopes in 2021 and the highest percentage in 2015-2019.

[e] p value associated with the comparison between the percentage of myopes in 2020 and the averaged percentage among 2015-2019.

[f] Z statistic value associated with the comparison between the percentage of myopes in 2020 and the averaged percentage among 2015-2019.

[g] p value associated with the comparison between the percentage of myopes in 2021 and the averaged percentage among 2015-2019.

[h] Z statistic value associated with the comparison between the percentage of myopes in 2021 and the averaged percentage among 2015-2019.

[i] p value associated with the comparison between the percentage of myopes in 2020 and the averaged percentage among 2015-2019 for all ages.

[j] Z statistic value associated with the comparison between the percentage of myopes in 2020 and the averaged percentage among 2015-2019 for all ages.

[k] p value associated with the comparison between the percentage of myopes in 2021 and the averaged percentage among 2015-2019 for all ages.

[l] Z statistic value associated with the comparison between the percentage of myopes in 2021 and the averaged percentage among 2015-2019 for all ages.

[m] Highest percentage of myopia relative to each age group, within the year range 2015-2019.

For peer review only

Supplemental table 3. Sample sizes as a function of age, gender, and year.

Age (years)	Gender	2015	2016	2017	2018	2019	2020	2021
6	female	394	407	433	427	464	355	363
	male	375	457	396	365	402	377	364
7	female	482	424	417	441	373	399	375
	male	384	399	396	385	390	324	351
8	female	453	439	460	403	423	440	407
	male	369	393	417	370	372	385	369
9	female	476	453	466	425	448	446	453
	male	400	380	375	360	371	368	358
10	female	573	534	513	521	512	464	464
	male	424	439	388	377	360	339	364
11	female	537	503	510	473	533	474	450
	male	413	407	348	345	343	337	336
12	female	534	567	564	509	533	523	472
	male	367	374	354	317	344	295	315
13	female	589	520	619	553	512	542	483
	male	356	349	373	341	335	314	306
14	female	568	552	554	607	541	475	465
	male	405	356	394	347	347	314	337
15	female	563	642	633	591	567	500	515
	male	399	374	381	362	349	348	301

For peer review only

STROBE 2007 (v4) Statement—Checklist of items that should be included in reports of *cross-sectional studies*

Section/Topic	Item #	Recommendation	Reported on page #
**Title and abstract**	1	(*a*) Indicate the study's design with a commonly used term in the title or the abstract	2, 3
		(*b*) Provide in the abstract an informative and balanced summary of what was done and what was found	3
**Introduction**			
Background/rationale	2	Explain the scientific background and rationale for the investigation being reported	5
Objectives	3	State specific objectives, including any prespecified hypotheses	5
**Methods**			
Study design	4	Present key elements of study design early in the paper	5-7
Setting	5	Describe the setting, locations, and relevant dates, including periods of recruitment, exposure, follow-up, and data collection	5-7
Participants	6	(*a*) Give the eligibility criteria, and the sources and methods of selection of participants	5-7
Variables	7	Clearly define all outcomes, exposures, predictors, potential confounders, and effect modifiers. Give diagnostic criteria, if applicable	5-7
Data sources/ measurement	8*	For each variable of interest, give sources of data and details of methods of assessment (measurement). Describe comparability of assessment methods if there is more than one group	5-7
Bias	9	Describe any efforts to address potential sources of bias	5-7
Study size	10	Explain how the study size was arrived at	5-7
Quantitative variables	11	Explain how quantitative variables were handled in the analyses. If applicable, describe which groupings were chosen and why	5-7
Statistical methods	12	(*a*) Describe all statistical methods, including those used to control for confounding	5-7
		(*b*) Describe any methods used to examine subgroups and interactions	5-7
		(*c*) Explain how missing data were addressed	5-7
		(*d*) If applicable, describe analytical methods taking account of sampling strategy	5-7
		(*e*) Describe any sensitivity analyses	5-7
**Results**			

For peer review only

Participants	13*	(a) Report numbers of individuals at each stage of study—eg numbers potentially eligible, examined for eligibility, confirmed eligible, included in the study, completing follow-up, and analysed	7-11
		(b) Give reasons for non-participation at each stage	7-11
		(c) Consider use of a flow diagram	7-11
Descriptive data	14*	(a) Give characteristics of study participants (eg demographic, clinical, social) and information on exposures and potential confounders	7-11
		(b) Indicate number of participants with missing data for each variable of interest	7-11
Outcome data	15*	Report numbers of outcome events or summary measures	7-11
Main results	16	(*a*) Give unadjusted estimates and, if applicable, confounder-adjusted estimates and their precision (eg, 95% confidence interval). Make clear which confounders were adjusted for and why they were included	7-11
		(*b*) Report category boundaries when continuous variables were categorized	7-11
		(*c*) If relevant, consider translating estimates of relative risk into absolute risk for a meaningful time period	7-11
Other analyses	17	Report other analyses done—eg analyses of subgroups and interactions, and sensitivity analyses	7-11
**Discussion**			
Key results	18	Summarise key results with reference to study objectives	12-15
Limitations	19	Discuss limitations of the study, taking into account sources of potential bias or imprecision. Discuss both direction and magnitude of any potential bias	15
Interpretation	20	Give a cautious overall interpretation of results considering objectives, limitations, multiplicity of analyses, results from similar studies, and other relevant evidence	12-15
Generalisability	21	Discuss the generalisability (external validity) of the study results	12-15
**Other information**			
Funding	22	Give the source of funding and the role of the funders for the present study and, if applicable, for the original study on which the present article is based	17

*Give information separately for cases and controls in case-control studies and, if applicable, for exposed and unexposed groups in cohort and cross-sectional studies.

Note: An Explanation and Elaboration article discusses each checklist item and gives methodological background and published examples of transparent reporting. The STROBE checklist is best used in conjunction with this article (freely available on the Web sites of PLoS Medicine at http://www.plosmedicine.org/, Annals of Internal Medicine at http://www.annals.org/, and Epidemiology at http://www.epidem.com/). Information on the STROBE Initiative is available at www.strobe-statement.org.

For peer review only
